# Emergency Management of Diffuse Large B-cell Lymphoma: A Case of Superior Vena Cava Syndrome and Chylothorax

**DOI:** 10.7759/cureus.97953

**Published:** 2025-11-27

**Authors:** Soraia G Araújo, Inês M Araújo, Margarida Robalo, Sofia Esperança, Ana Oliveira

**Affiliations:** 1 Critical Care Medicine, Hospital de Braga, Braga, PRT; 2 Internal Medicine, Hospital de Braga, Braga, PRT

**Keywords:** diffuse large b-cell lymphoma, lymphoma, malignant chylothorax, mediastinal mass, superior vena cava syndrome

## Abstract

Diffuse large B-cell lymphoma (DLBCL) is the most common subtype of non-Hodgkin lymphoma and often presents with an aggressive clinical course. We report the case of an 84-year-old woman with dementia who presented with a rapidly growing mediastinal mass. Imaging revealed compression of the superior vena cava - an oncological emergency requiring immediate intervention - along with a large pleural effusion, which was confirmed to be a chylothorax, a rare complication of lymphoma. Radiotherapy was started on the day of admission, alongside corticosteroid therapy. A biopsy confirmed peripheral B-cell lymphoma, consistent with stage IE DLBCL. Despite her advanced age, dementia, and other comorbidities, palliative chemotherapy was initiated. This case underscores the challenges of managing aggressive lymphoma in older patients with significant comorbidities and highlights the importance of prompt diagnosis and targeted treatment.

## Introduction

Diffuse large B-cell lymphoma (DLBCL) is the most prevalent subtype of non-Hodgkin lymphoma, accounting for 30-58% of cases across various populations worldwide [1,2]. It exhibits a broad clinical spectrum, typically presenting as a rapidly enlarging mass involving one or more lymph nodes, and may also involve extranodal sites, most commonly the gastrointestinal tract [2]. DLBCL is an aggressive malignancy, often seen in older adults, particularly those in their seventh decade of life [2]. In Europe, the incidence is approximately 3.8 cases per 100,000 individuals per year [1].

DLBCL has multiple clinical presentations, and the prognosis is influenced by the primary site of involvement, the patient's age, and overall clinical condition. The clinical presentation of DLBCL ranges from nonspecific symptoms, such as fatigue and weight loss, to more emergent findings, such as superior vena cava (SVC) syndrome or gastrointestinal hemorrhage, depending on the size and anatomical location of the tumor [2]. A rare complication of DLBCL is compression or invasion of the thoracic duct, resulting in accumulation of lymphatic fluid within the pleural space, a condition known as chylothorax [3]. The simultaneous occurrence of SVC syndrome and chylothorax at presentation is uncommon, further highlighting the clinical significance of such cases.

While early identification is critical, as prompt initiation of treatment is associated with better outcomes, in some cases, particularly in older patients or those with multiple comorbidities, management may primarily focus on symptom control. We present a case of an elderly woman with a rapidly growing mediastinal mass causing rare and life-threatening manifestations, such as SVC syndrome. This case represents a diagnostic and therapeutic challenge in the face of extensive comorbid disease.

## Case presentation

An 84-year-old woman was brought to the emergency department following an episode of dyspnea, peripheral cyanosis, and oxygen desaturation. A more detailed anamnesis revealed that this was not the first occurrence of such symptoms. In fact, for approximately one month, the patient had experienced progressive dyspnea and edema of the face and neck, which were exacerbated in the supine position. These symptoms worsened over time, ultimately rendering the patient unable to tolerate lying flat and needing to sleep in the seated position. Her medical history included hypertension, dyslipidemia, and advanced dementia.

On physical examination, the patient was observed in a semi-recumbent position with the head of the bed elevated to approximately 35 degrees. Vital signs revealed a blood pressure of 128/54 mmHg, a heart rate of 90 bpm in sinus rhythm, a respiratory rate of 31 breaths per minute, a temperature of 97.3° F, and an oxygen saturation of 86% on room air. Facial and neck edema were observed, with negative Godet’s sign, along with evidence of venous collateral circulation over the chest wall. Jugular venous distention was observed, and no tracheal deviation was noted. No palpable lymphadenopathy was identified. Pulmonary auscultation revealed absent breath sounds in the lower two-thirds of the right lung field and bilateral wheezing. The remainder of the physical examination was unremarkable.

**Figure 1:**
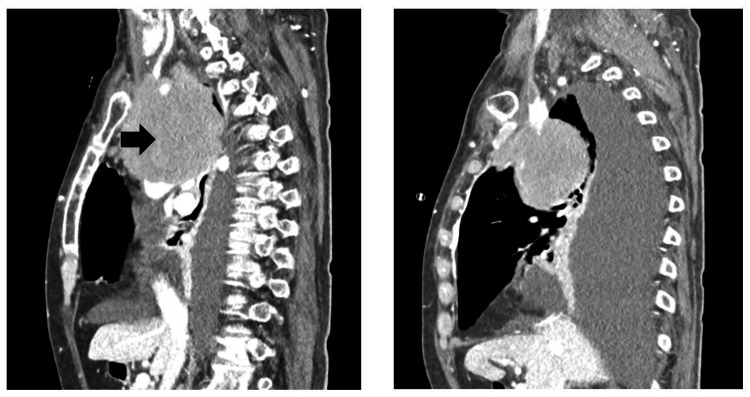
Large mediastinal mass (black arrow) involving the paratracheal, pre- and infracarinal spaces, and aortopulmonary window, extending into the anterior mediastinum, surrounding the brachiocephalic trunk and the origin of the left common carotid artery (without occlusion or arterial stenosis). The mass measures 79 x 87 mm in its largest axes in the transverse plane.

**Figure 2:**
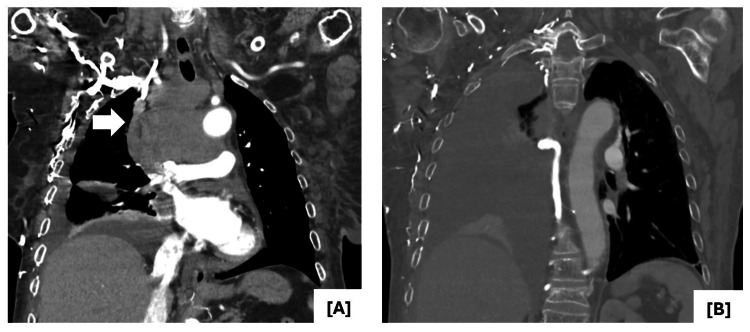
A) Obliteration of the middle third of the superior vena cava, subclavian vein, and distal portion of the right jugular vein (white arrow). Presence of collateral circulation in the right chest wall, associated with the previously mentioned vascular occlusions. B) Large-volume right pleural effusion.

**Figure 3:**
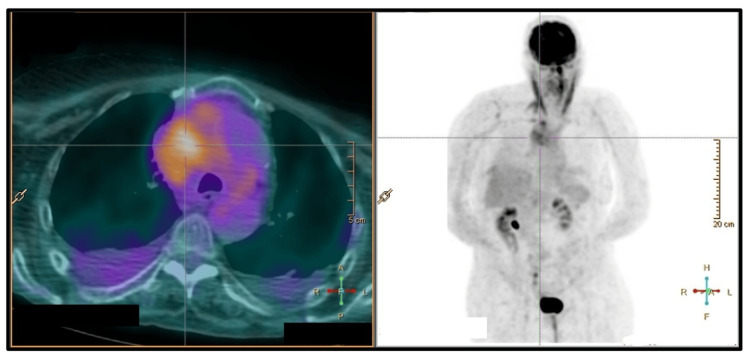
PET scan reveals mass with ill-defined limits in the anterior mediastinum, laterally displaced to the right, extending into the prevascular and paratracheal planes, inseparable from adjacent vessels and airways, with heterogeneous uptake of the radiopharmaceutical, suggestive of a hypermetabolic malignant tumor.

Laboratory investigations revealed lymphopenia, elevated lactate dehydrogenase, and elevated beta-2 microglobulin (Table 1). Contrast-enhanced computed tomography (CT) of the chest presented a large mediastinal mass (Figure 1) compressing the SVC and right innominate vein, along with a sizable right pleural effusion (Figure 2). Positron emission tomography (PET) scan demonstrated a hypermetabolic malignant lesion in the anterior mediastinum (Figure 3), with no evidence of distant metastatic uptake.

**Table 1:** Initial laboratory workup MCV: mean corpuscular volume; MCHC: mean corpuscular hemoglobin concentration; AST: aspartate aminotransferase; ALT: alanine aminotransferase; ALP: alkaline phosphatase; LDH: lactate dehydrogenase

Laboratory workup parameters	Values	Reference values
Hemoglobin	12.00 g/dL	11.9-15.6 g/dL
MCV	88.8 fL	82.9-98.0 fL
MCHC	32.20 g/dL	31.8-34.7 g/dL
Leukocytes	4700 /uL	4000-11000 /µL
Neutrophils	4200 /uL	1800-7100 /µL
Lymphocytes	200 /uL	1200-3400 /µL
Monocytes	300 /uL	200-900 /µL
Platelets	222000 /uL	150000-400000 /µL
Urea	45 mg/dL	19-49 mg/dL
Creatinine	0.90 mg/dL	0.6-1.1 mg/dL
Potassium	3.80 mmol/L	3.5-5.5 mmol/L
Sodium	137 mmol/L	135-145 mmol/L
Total bilirubin	0.40 mg/dL	0.2-1.1 mg/dL
AST	46 U/L	12-40 U/L
ALT	46 U/L	7-40 U/L
ALP	68 U/L	46-116 U/L
LDH	347 U/L	120-246 U/L
C-reactive protein	22.50 mg/L	<5 mg/L
Beta-2 microglobulin	4285 ng/mL	1000-2400 ng/mL

**Figure 4:**
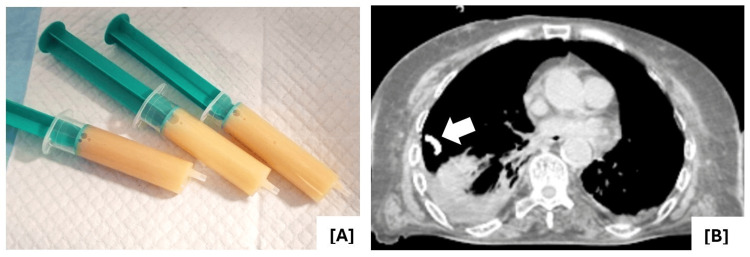
A) Thick, yellowish pleural fluid aspirated during thoracentesis. B) Pigtail chest tube (white arrow) positioned at the level of the fourth intercostal space, along the right mid-axillary line.

Ultrasound-guided diagnostic thoracentesis was performed, yielding a thick, yellowish fluid (Figure 4), with biochemical characteristics consistent with chylothorax (triglyceride level of 700 mg/dL). Microbiological, mycological, and cytological analysis of the fluid were negative. Given the substantial volume of the chylothorax, a right-sided chest tube was placed (Figure 4), with a total drainage of approximately 4500 mL over six days. The daily output was initially high (up to 1,000 mL/day) but progressively decreased throughout hospitalization, consistent with partial resolution after initial therapeutic measures.

Given the radiological appearance of the mediastinal mass, which was suggestive of a malignant process, a CT-guided transthoracic biopsy was performed. Considering the high suspicion of lymphoproliferative disorder, a five-day course of prednisolone (1 mg/kg/day) and prophylaxis for tumor lysis syndrome (hydration and allopurinol) were initiated. As the mass was causing SVC syndrome due to compression of mediastinal structures - constituting an oncologic emergency - the case was immediately discussed with the radiation oncology team, and the first of five radiotherapy sessions was administered on the day of admission.

**Figure 5:**
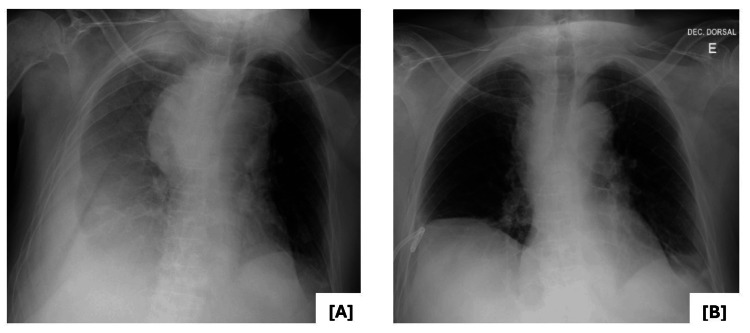
Chest X-ray A) before and B) after chest tube placement, completion of corticosteroid therapy, and radiotherapy sessions. Actual scale unavailable due to image export limitations.

Following completion of corticosteroid therapy and radiotherapy, follow-up chest radiography demonstrated resolution of the pleural effusion and significant reduction of the mediastinal mass (Figure 5).

Immunohistochemical analysis of the biopsied mass revealed a polymorphic lymphoid population with diffuse expression of CD20, PAX-5, BCL-2, and CD10; multifocal (>30%) expression of BCL-6; and focal expression of CD30, MUM1, and CD23, with rare expression of c-Myc. These findings established the diagnosis of peripheral B-cell lymphoma, consistent with stage IE DLBCL. Representative immunohistochemical images were unavailable for publication; however, the findings are described in detail based on the official pathology report.

Given the patient's advanced age and multiple comorbidities, curative treatment was deemed unfeasible. After a multidisciplinary team discussion, a decision was made to initiate palliative therapy with an oral chemotherapy regimen consisting of prednisone, etoposide, procarbazine, and cyclophosphamide (PEP-C).

## Discussion

DLBCL is a non-Hodgkin lymphoma that is aggressive and frequently develops rapidly, presenting with a variety of clinical signs and symptoms [2]. Although it can also affect other areas, such as the gastrointestinal system, lungs, and mediastinum, DLBCL is usually identified as a developing mass in lymph nodes [2]. In this case, the patient presented with a mediastinal mass accompanied by symptoms of SVC syndrome and chylothorax, highlighting the urgency and complexity associated with the diagnosis and treatment of this type of lymphoma.

The symptoms of SVC syndrome arise from obstruction or invasion of the SVC with various presentations, including edema of the face and upper extremities, dyspnea, venous distention, and potentially cyanosis and altered level of consciousness if severe. Life-threatening symptoms that require immediate intervention may include neurological deficits, stridor, or hemodynamic instability [4]. Most diagnoses of SVC syndrome are made using imaging; usually, a contrast-enhanced CT scan is ordered and may show a compressive or infiltrative mass within the SVC territory [4]. An acute SVC syndrome clinical diagnosis requires the bed to be raised, supplemental oxygen, and corticosteroids only if a lymphoma is likely to be steroid-sensitive, and an expedited oncology evaluation [4]. If tumor thrombosis is suspected, anticoagulation or thrombolysis may be required. Being aware that all mediastinal masses are not lymphomas is necessary (other considerations include studies that could include lung cancer, thymoma, germ cell tumors, or non-neoplastic processes, e.g., thrombosis, fibrosing mediastinitis) [4]. Biopsy of the lesion is necessary for diagnostic confirmation and may require rapid tumor reduction for treatment. Notably, acute treatment of malignancy may include corticosteroids, potentially life-saving chemotherapy, and radiation. Depending on the indication, SVC stenting can be considered for refractory SVC syndrome or immediate control of symptoms [4].

Chylothorax is a rare complication of DLBCL and usually occurs due to compression or invasion of the thoracic duct. It results in chyle accumulation in the pleural space and both respiratory and systemic complications [3]. Beyond respiratory compromise, high-output chylothorax (as seen in this patient with 4500 mL drained over six days) can cause hypoproteinemia, lymphopenia with immunosuppression, electrolyte imbalance, and malnutrition [3]. Prolonged drainage can lead to dehydration and, in some cases, a persistent lymphatic fistula that may require pleurodesis or surgery. Management includes careful control of drainage volume, nutritional support (e.g., a diet rich in medium-chain triglycerides or parenteral nutrition), and fluid/electrolyte replacement [3]. The primary goal is to treat the underlying malignancy. In refractory or persistent cases, surgical options like thoracic duct ligation or pleurodesis may be needed, and watch for infection [3].

In this case, the imaging studies (which encompass CT and PET scans) proved critical in diagnosing a mediastinal mass and confirming the malignant nature of the lesion; these diagnostic tools guided the decision to initiate radiotherapy immediately upon admission - an essential step in managing SVC syndrome and reducing tumor burden. This approach is consistent with current emergency oncology practice and international guidelines for SVC syndrome. Corticosteroid therapy was also started, along with measures to prevent tumor lysis syndrome, which often complicates the management of patients with high-grade hematological malignancies such as B-cell non-Hodgkin lymphoma [5].

The diagnosis of DLBCL was established via biopsy and immunohistochemistry, consistent with stage IE DLBCL. Given the patient's advanced age and the degree of cognitive impairment associated with her dementia, she was not suitable for curative therapy using conventional approaches such as R-CHOP (rituximab, cyclophosphamide, doxorubicin, vincristine, prednisolone). Although R-CHOP is effective, it carries substantial risks for toxicities associated with myelosuppression, cardiotoxicity, and infection that are likely outweighed by the potential benefits for this frail patient [1]. Instead, a palliative chemotherapy regimen (PEP-C) was initiated. PEP-C is an oral metronomic therapy that is more tolerable for elderly or frail patients and has previously shown clinically significant symptom control and improved quality of life, and less associated toxicity in select instances [6].

This case illustrates several important aspects of DLBCL management, especially in older patients with significant morbidities. Treatment planning requires consideration of prognosis, treatment tolerance, and the patient’s baseline functional and cognitive status. The significance of early identification and proper treatment for oncologic emergencies is essential, and a multidisciplinary approach is particularly valuable when curative therapies are not feasible.

Recognition of significant complications such as SVC syndrome and chylothorax should be prompt, as timely intervention can improve outcomes. In geriatric lymphoma care, individualized and compassionate management, balancing therapeutic benefit with quality of life, often becomes the central focus.

## Conclusions

This case highlights the value of early diagnosis and intervention in the management of DLBCL, principally with complications such as SVC syndrome and chylothorax. Considering the aggressive nature of DLBCL, as well as the patient’s age and comorbidities, it was necessary to approach the case with attention to prognosis, expected treatment tolerance, and overall quality of life. Despite her dementia and other medical conditions, these factors supported the early initiation of radiation therapy, together with corticosteroid treatment and subsequent palliative chemotherapy, which contributed to symptom control and improved comfort. In caring for elderly patients with DLBCL, customized compassionate care may become more important than treatment aimed at curing the disease itself, focusing instead on symptom management and supportive care. More importantly, the case underlines the value of timely diagnosis and targeted management of oncologic emergencies in the geriatric population.
